# The Morphological, Thermal, and Mechanical Properties of Glass Ionomer Modified Polylactic Acid Polymer

**DOI:** 10.1155/ijod/4603534

**Published:** 2026-08-30

**Authors:** Rungroj Kuson, Thanapat Sastraruji, Kullapop Suttiat

**Affiliations:** ^1^ Department of Prosthodontics, Faculty of Dentistry, Chiang Mai University, Chiang Mai 50200, Thailand, cmu.ac.th; ^2^ Dental Research Center, Faculty of Dentistry, Chiang Mai University, Chiang Mai 50200, Thailand, cmu.ac.th

**Keywords:** dental materials, glass ionomer cement (GIC), polylactic acid (PLA), polymer composites, sodium fluoride (NaF)

## Abstract

This study evaluated the morphological, thermal, and mechanical behavior of polylactic acid (PLA) composites modified with conventional self‐cured glass‐ionomer cement (GIC) (Fuji VII; GC Corporation, Japan)—fully set and ground into powder—or sodium fluoride (NaF; Merck, USA) under simulated oral hydrolytic degradation. PLA based films containing 0–20 wt% fillers were produced by solvent casting, immersed in phosphate‐buffered saline (PBS) at 37°C for up to 28 days, and characterized using scanning electron microscopy, differential scanning calorimetry, and tensile testing. Surface analysis showed that GIC filled PLA maintained a slight roughness and intact morphology with minimal void formation over time, whereas high NaF loading promoted pronounced surface porosity due to filler dissolution and enhanced water ingress. Thermal analysis indicated small increases in glass transition temperature after filler addition and aging, while cold crystallization and melting enthalpies were strongly dependent on filler type and concentration, with 5 wt% GIC supporting high crystallinity and higher loadings progressively suppressing crystal development during aging. Mechanically, pure PLA displayed an initial increase in tensile strength followed by marked deterioration, NaF filled films exhibited concentration dependent weakening particularly at 20 wt%, and PLA containing 5 wt% GIC showed the most stable tensile strength response over 28 days. Overall, PLA containing a low content of GIC, particularly 5 wt%, provides a favorable balance of structural stability and controlled crystallization. These findings warrant further preclinical investigation as a candidate fluoride‐releasing and rechargeable material for dental applications. This study does not establish clinical suitability.

## 1. Introduction

The use of removable partial dentures (RPDs) among partially edentulous adults continues to rise across many populations [1], yet RPDs can create plaque‐retentive environments that facilitate persistent biofilm accumulation and caries progression [2].

Fluoride, a well‐established anticariogenic agent acting through enamel remineralization, demineralization inhibition, and antimicrobial effect [3], is nonetheless limited at the tooth surface by rapid clearance from the oral cavity, which substantially reduces its protective duration [4, 5]. This limitation is particularly relevant for RPD wearers, whose prosthetic surfaces create persistent, localized caries risk [1] that conventional fluoride regimens may not adequately address. Although self‐applied fluoride products such as toothpastes and mouth rinses remain effective for the general population [6, 7], elderly RPD wearers frequently present with reduced manual dexterity, cognitive decline, and impaired self‐care ability [8–10], which can compromise the consistent and effective use of these conventional regimens. Sustained passive fluoride delivery embedded within the prosthesis itself may therefore offer a compliance‐independent alternative that is less reliant on patient adherence [11]. Prosthetic materials capable of sustained fluoride release therefore offer a promising strategy to overcome this limitation, with fluoride‐containing denture materials emerging as an approach for maintaining prolonged intraoral fluoride availability [12].

Towards this goal, previous investigations have demonstrated that heat‐polymerized polymethyl methacrylate (PMMA) denture bases incorporating sodium fluoride (NaF) or surface pre‐reacted glass‐ionomer (S‐PRG) fillers at approximately 20 wt% can function as effective fluoride reservoirs [13, 14]. However, direct incorporation of fluoride‐releasing particles into the denture base polymer matrix has been reported to adversely affect physical [15] and mechanical properties [14], representing a persistent challenge for both clinical application and materials design.

As an alternative that avoids modifying the denture base itself, our previous in vitro investigation demonstrated that a newly developed polylactic acid/glass‐ionomer cement (PLA/GIC) composite film exhibited sustained fluoride release at levels conducive to tooth remineralization for up to 28 days, together with fluoride recharge capability following exposure to NaF solution [16].

While these fluoride‐related findings are promising, clinical application also depends on the overall performance of the polymer itself, which is governed by its inherent nature and crystallinity, as well as by the quantity and characteristics of the incorporated filler—including morphology, volume fraction, reactivity, surface area, melting temperature, and oxygen concentration [17–19]. Among these factors, the glass transition temperature (*T*_g_) is particularly critical, as key physical properties of PLA—including density, heat capacity, and mechanical and rheological behavior—are closely linked to *T*_g_ [20], which itself represents a major limitation to the broader clinical application of PLA composites [21].

Given that alterations in morphological, thermal, and mechanical properties are critical determinants of clinical feasibility and long‐term material stability, yet remain insufficiently characterized in fluoride‐releasing systems, such as PLA/GIC, the present study aimed to investigate these changes in a newly developed PLA/GIC composite following simulated hydrolytic degradation, with PLA/NaF films included for comparison. We hypothesized that filler type, filler concentration, and aging time would significantly affect the morphological, thermal, and mechanical properties of the PLA films against the corresponding null hypothesis of no such effects.

## 2. Materials and Methods

### 2.1. Materials

PLA (PLA 4043D) was obtained from NatureWorks (Plymouth, MN, USA) and used as the polymer matrix. NaF was supplied by Merck (Rahway, NJ, USA), and GIC (Fuji VII) was provided by GC Corporation (Tokyo, Japan). Phosphate‐buffered saline (PBS) was purchased from Sigma–Aldrich (St. Louis, MO, USA), and chloroform was obtained from Union Science (Chiang Mai, Thailand). All chemicals and reagents were used as received without any additional purification.

### 2.2. Sample Preparation

PLA‐based composite films were fabricated using the solvent‐casting technique. PLA pellets were first dissolved in chloroform at a concentration of 10% (*w*/*v*) by continuous magnetic stirring at room temperature for 8 h until a clear and homogeneous solution was obtained. The self‐cured GIC, consisting of a fluoroaluminosilicate glass powder and an aqueous polyacrylic acid liquid, was prepared according to the manufacturer’s instructions and allowed to set via an acid–base reaction [22, 23]. The set cement was then ground using a mortar and pestle, and the resulting GIC and NaF powders were sieved twice through laboratory test sieves (1600 mesh) to obtain a uniform particle size.

To characterize particle size, the sieved powders were mounted on aluminum stubs, sputter‐coated with a thin gold layer, and imaged by scanning electron microscopy (SEM; TESCAN VEGA4, Brno, Czech Republic) at 5000× magnification and an accelerating voltage of 10 keV. Particle diameters were measured from 10 randomly selected particles across five representative micrographs for each powder type, yielding uniform mean particle sizes of 1.11 ± 0.07 µm for GIC and 0.81 ± 0.02 µm for NaF.

**Figure 1:**
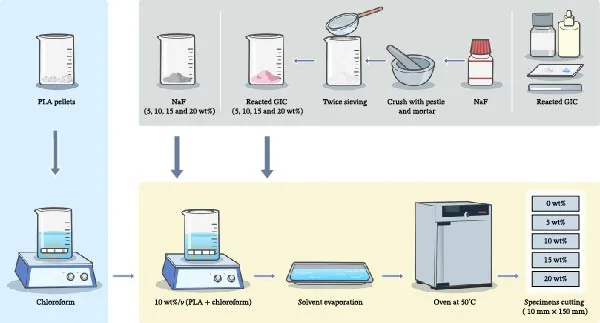
A schematic illustration of specimen preparation.

The particles were then incorporated into the PLA/chloroform solution at concentrations of 0, 5, 10, 15, and 20 wt%. Each composite solution was magnetically stirred at room temperature for an additional 1 h to ensure uniform dispersion of the additive particles within the polymer matrix [16]. A total volume of 150 mL of each prepared solution was poured into a stainless‐steel casting tray with dimensions of 38.3 cm × 26.5 cm × 1.9 cm. Solvent evaporation was carried out at room temperature under a fume hood for 24 h. The resulting films were further dried in a temperature‐ and humidity‐controlled chamber at 50°C for 8 h to remove the residual solvent [16]. After cooling to room temperature, the films were cut into rectangular specimens measuring 10 mm × 150 mm, in accordance with ISO 527‐3:2018, for subsequent characterization, as presented in Figure 1.

### 2.3. Sample Characterization

For each material formulation, 10 specimens of PLA/NaF and PLA/GIC composite films containing additive particles at concentrations of 0%, 5%, 10%, 15%, and 20% by weight were prepared. Each formulation was divided into two subgroups to evaluate material properties before and after exposure to a simulated oral degradative environment in accordance with ISO 15814.

Specimens in the first subgroup (*n* = 5) were evaluated immediately after fabrication to determine baseline morphological, thermal, and mechanical properties. Specimens in the second subgroup (*n* = 5 at each time point) were subjected to simulated oral hydrolytic degradation by individual immersion in 30 mL of PBS and incubation at 37°C for periods of 7, 14, 21, and 28 days. At each designated time point, specimens were retrieved, gently rinsed with distilled water, blotted with absorbent paper to remove surface moisture, and dried at room temperature for 24 h. The dried specimens were subsequently subjected to the same morphological, thermal, and mechanical characterization procedures as those applied to the baseline subgroup.

#### 2.3.1. Surface Morphology

The specimens (air‐exposed side) were sputter‐coated with a thin gold layer to enhance the surface conductivity. Surface morphology of the PLA/NaF and PLA/GIC composite films was examined using a scanning electron microscope (SEM; TESCAN VEGA4, Brno, Czech Republic) at 5000x with an accelerating voltage of 10 keV.

#### 2.3.2. Thermal Property

Thermal and crystallization behaviors of the PLA/NaF and PLA/GIC composite films were analyzed using a differential scanning calorimeter (DSC1, Mettler‐Toledo, Australia). Approximately 5 mg of each specimen was sealed in an aluminum pan and subjected to a heating–cooling–reheating protocol. Samples were first heated from 30 to 190°C at a rate of 10°C/min and held at 190°C for 3 min to erase the prior thermal history. Subsequently, samples were cooled to 30°C at the same rate, followed by a second heating cycle from 30 to 190°C at 10°C/min [24].

#### 2.3.3. Mechanical Property

Tensile properties of the PLA‐based composite films before and after simulated artificial hydrolytic degradation were determined using a universal testing machine (AUTOGRAPH AGX‐V Series, Shimadzu, Kyoto, Japan). Mechanical testing was conducted at a crosshead speed of 50 mm/min in accordance with ISO 527‐3:2018 for plastic films and sheets.

### 2.4. Statistical Analysis

Statistical analyses were performed using the IBM SPSS Statistics software (version 25; IBM Corporation, New York, NY, USA). Two‐way analysis of variance (ANOVA) was used to evaluate the effects of additive type (PLA/NaF and PLA/GIC) and additive concentration on the tensile strength of the composite films. When significant differences were detected, post hoc comparisons were performed using Tukey’s honestly significant difference (HSD) test. A *p*‐value of less than 0.05 was considered statistically significant.

## 3. Results

### 3.1. Surface Morphology

SEM images illustrating the surface morphology of pure PLA, PLA/NaF, and PLA/GIC composite films with varying additive concentrations at different stages of in vitro degradation are presented in Figure 2. Prior to artificial hydrolytic degradation, pure PLA exhibited a smooth and uniform surface. Incorporation of NaF or GIC particles into the PLA matrix resulted in a relatively rougher surface texture. No visible fracture lines or porous structures were observed on the surfaces of any specimens at this stage. Following hydrolytic degradation, surface roughening accompanied by the formation of small voids was observed in the pure PLA group. However, no pronounced or progressive surface changes were detected throughout the degradation period. Among the PLA/NaF composites, the specimen with 20 wt% NaF showed the most pronounced void formation after degradation, whereas specimens with lower NaF concentrations exhibited only minimal surface changes over the same testing periods. In contrast, PLA/GIC composite films displayed no notable differences in the surface morphology across the range of additive concentrations at each degradation time point.

Figure 2Representative SEM micrographs of pure PLA. (a) PLA/NaF and (b) PLA/GIC films at baseline and after immersion in PBS (37°C) for up to 28 days.

**Figure:**
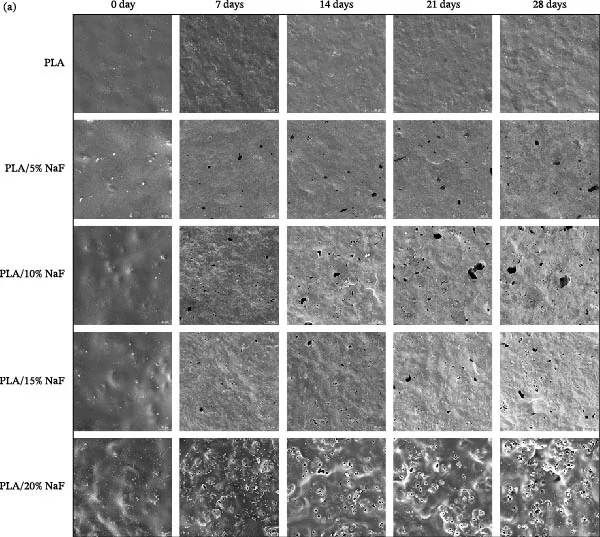

**Figure:**
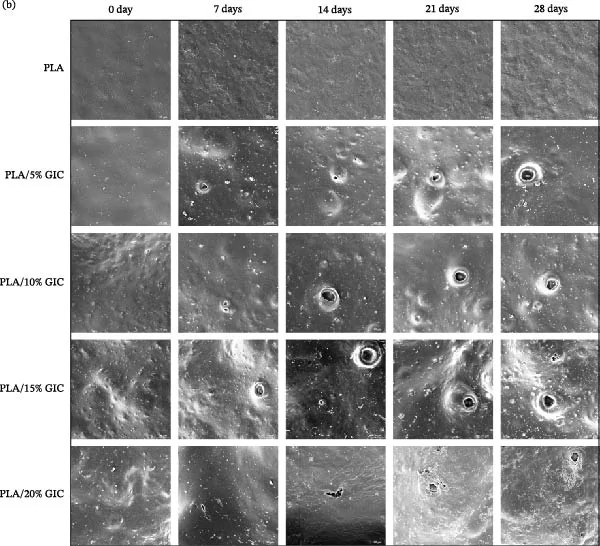

### 3.2. Thermal Properties

**Figure 3:**
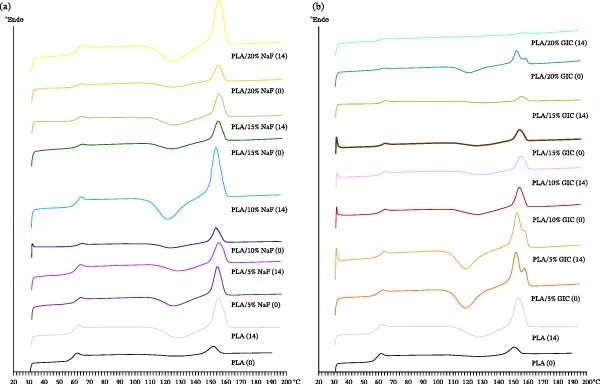
DSC thermograms (second heating cycle) of pure PLA. (a) PLA/NaF and (b) PLA/GIC at baseline and after 14 days of PBS immersion (37°C).

The thermal behavior of pure PLA, PLA/NaF, and PLA/GIC films was evaluated using differential scanning calorimetry (DSC), with the glass transition temperature (*T*_g_), cold crystallization enthalpy (Δ*H*_c_), and melting enthalpy (Δ*H*_m_) determined from the second heating cycle. Thermal measurements were performed after 0 and 14 days of in vitro degradation, as presented in Figure 3 and Table 1. Measurements were obtained from a single specimen per group at each time point owing to the time‐intensive nature of the heating–cooling–reheating protocol required for each run and budgetary constraints associated with DSC analysis costs. Consequently, the following *T*_g_, Δ*H*_c_, and Δ*H*_m_ values are reported as descriptive trends rather than statistically validated differences, and this represents an acknowledged limitation of the present study that future work should address through replicate measurements.

**Table 1:** Thermal properties (*T*_g_, ∆*H*_c_, and ∆*H*_m_) of pure PLA and PLA‐based composite films at baseline and after 14 days of PBS immersion (37°C).

Group	Time (days)					
	0 day			14 days		
	*T*_g_ (°C)	∆*H*_c_ (J/g)	∆*H*_m_ (J/g)	*T*_g_ (°C)	∆*H*_c_ (J/g)	∆*H*_m_ (J/g)
PLA	57.10	3.04	3.10	56.67	15.74	13.69
PLA/5% NaF	57.74	11.83	11.26	57.71	8.43	9.27
PLA/10% NaF	58.11	5.06	6.61	58.28	21.20	24.40
PLA/15% NaF	58.61	7.55	8.33	58.16	10.01	10.59
PLA/20% NaF	58.26	5.73	6.51	58.08	20.67	20.32
PLA/5% GIC	57.51	18.86	19.32	58.31	19.81	17.31
PLA/10% GIC	58.44	6.98	7.81	58.51	4.75	5.27
PLA/15% GIC	58.19	3.47	5.03	58.70	1.39	1.92
PLA/20% GIC	57.16	4.90	6.55	58.90	ND^a^	0.34

^a^Not detected under the applied DSC conditions.

At the initial time point (0 day), the glass‐transition temperature (*T*_g_) of all groups ranged from 57.1 to 58.61°C. Pure PLA exhibited a *T*_g_ of 57.1°C, while incorporation of NaF or GIC particles resulted in a modest increase in *T*_g_. The highest *T*_g_ value was observed in PLA containing 15 wt% NaF (58.61°C), followed by PLA containing 10 wt% GIC (58.44°C). After 14 days of in vitro degradation, minor fluctuations in *T*_g_ were observed among the tested groups. Pure PLA showed a slight decrease in *T*_g_ to 56.67°C, whereas the composite films generally maintained or exhibited a modest increase in *T*_g_. Notably, PLA containing 20 wt% GIC demonstrated the highest *T*_g_ value after degradation (58.9°C).

The cold crystallization enthalpy (Δ*H*_c_) exhibited more pronounced changes during the degradation period compared with those of other thermal parameters. For neat PLA, Δ*H*_c_ increased markedly from 3.04 to 15.74 J/g after 14 days. In NaF‐filled composites, changes in Δ*H*_c_ appeared concentration‐dependent. PLA containing 5 wt% NaF showed a decrease in Δ*H*_c_ from 11.83 to 8.43 J/g. In contrast, composites with higher NaF contents (10, 15, and 20 wt%) exhibited substantial increases in Δ*H*_c_ over the aging period, rising from 5.06 to 21.20 J/g, from 7.55 to 10.01 J/g, and from 5.73 to 20.67 J/g, respectively. Conversely, PLA/GIC composites displayed a distinct thermal trend. PLA with 5 wt% GIC maintained a relatively high Δ*H*_c_, increasing slightly from 18.86 to 19.81 J/g. However, with increasing GIC content, Δ*H*_c_ appeared to progressively decrease from 6.98 to 4.75 J/g at 10 wt%, from 3.47 to 1.39 J/g at 15 wt%, and from 4.90 J/g at 20 wt% to values below the detection limit after degradation.

The melting enthalpy (Δ*H*_m_) exhibited pronounced formulation and time‐dependent changes. For pure PLA, Δ*H*_m_ increased substantially from 3.10 to 13.69 J/g after 14 days of degradation. In PLA/NaF composites, the initial Δ*H*_m_ values were higher than those of neat PLA across all NaF concentrations, measuring 11.26, 6.61, 8.33, and 6.51 J/g for 5, 10, 15, and 20 wt% NaF, respectively. After 14 days, PLA with 5% NaF showed a modest reduction in Δ*H*_m_ to 9.27 J/g, whereas composites with 10, 15, and 20 wt% NaF exhibited marked increases, reaching 24.40, 10.59, and 20.32 J/g, respectively. In contrast, PLA/GIC composites demonstrated a distinctly different trend. At day 0, Δ*H*_m_ values were 19.32, 7.81, 5.03, and 6.55 J/g for the 5, 10, 15, and 20 wt% GIC formulations, respectively. Following 14 days of degradation, Δ*H*_m_ decreased consistently with increasing GIC content, declining to 17.31 J/g for 5 wt% GIC, 5.27 J/g for 10 wt% GIC, 1.92 J/g for 15 wt% GIC, and 0.34 J/g for 20 wt% GIC.

### 3.3. Mechanical Property

**Figure 4:**
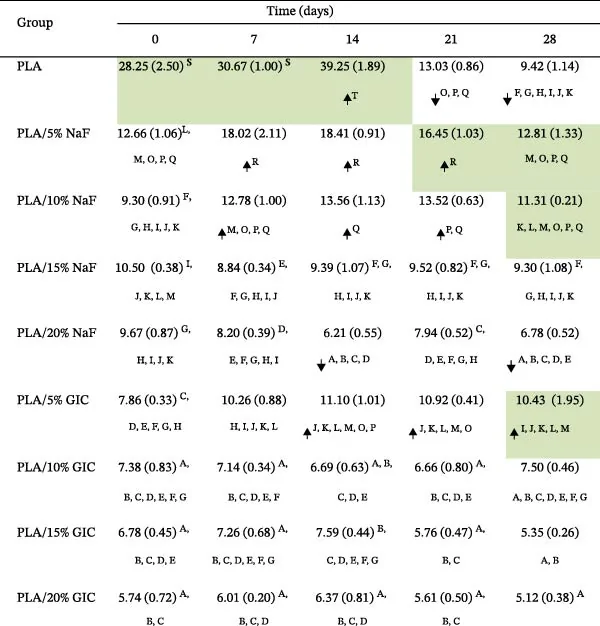
Tensile strength of pure PLA and PLA‐based composite films (MPa) after PBS immersion (37°C) for 0, 7, 14, 21, and 28 days. Values represent mean ± SD (*n* = 5 at each time point). Within each column (time point), values sharing at least one superscript letter are not significantly different (*p*  < 0.05)↑ and ↓ indicates a statistically significant increase or decrease, respectively, relative to the baseline (Day 0) value for that material (*p*  < 0.05); no symbol indicates no significant change from baseline. Shaded cells indicate the groups with the highest mean tensile strength at that time point and any other groups not significantly different from it.

The tensile strength of pure PLA, PLA/NaF, and PLA/GIC composite films exhibited distinct trends during the 4‐week degradation period, as presented in Figure 4. Pure PLA showed a significant increase in tensile strength during the second week, followed by a marked decline in the third and fourth weeks. The influence of NaF incorporation on tensile strength was concentration‐dependent. PLA containing 5 wt% NaF demonstrated a significant increase in tensile strength during the first week, followed by a notable decrease by the fourth week. PLA containing 10 wt% NaF also exhibited an initial improvement; however, the subsequent reduction was not statistically significant. In contrast, PLA containing 15 wt% NaF showed no significant changes in tensile strength throughout the experimental period. PLA containing 20 wt% NaF experienced a significant decrease in tensile strength during the second week, after which the values stabilized. GIC influenced the mechanical behavior of the composites in a different manner. PLA containing 5 wt% GIC showed a significant increase in tensile strength during the second week, followed by stabilization over the remaining time points. In contrast, composites containing 10, 15, and 20 wt% GIC did not exhibit statistically significant changes in tensile strength throughout the study period. Among all PLA/GIC formulations, the composite containing 5 wt% GIC consistently demonstrated the highest tensile strength over time.

## 4. Discussion

### 4.1. Surface Morphology

SEM analysis provided insight into the initial surface characteristics and early degradation behavior of pure PLA and PLA‐based composites. Prior to degradation, pure PLA exhibited a smooth and uniform surface, consistent with an intact and continuous polymer matrix [25]. Incorporation of particulate fillers, including NaF and GIC, resulted in increased surface roughness at baseline, reflecting the disruption of the originally smooth PLA surface by dispersed filler particles. This change is attributed to the presence of filler–matrix interfaces rather than structural damage [26], as no cracks, pores, or discontinuities were observed at the initial stage for either composite system.

During in vitro degradation, pure PLA showed only limited surface deterioration, characterized by mild roughening and the formation of small voids. These changes progressed slowly and remained modest throughout the observation period, indicating relatively homogeneous degradation under the applied conditions [27]. In contrast, the PLA composite containing 20 wt% NaF exhibited more pronounced surface degradation, including visible pores and irregular features. This behavior is consistent with increased water interaction at the material surface [27, 28] and localized dissolution of NaF particles, which are highly water soluble [29]. The resulting voids likely facilitated additional water ingress, promoting further hydrolytic degradation near the surface [30]. Although fluoride‐releasing fillers can introduce microstructural discontinuities that facilitate water penetration, the extent of this effect depended strongly on the filler type. PLA/GIC composites exhibited comparatively stable surface morphologies across all concentrations and degradation times. The absence of pronounced void formation suggests that cured GIC particles remained structurally intact within the PLA matrix [16] and did not undergo rapid dissolution [31, 32]. As a result, surface degradation in PLA/GIC composites was less pronounced than in high‐NaF formulations, indicating greater resistance to surface morphological changes under the tested conditions.

### 4.2. Thermal Properties

Thermal behavior was evaluated at baseline and after 14 days of in vitro degradation to assess early structural changes. Glass transition temperature (*T*_g_), cold crystallization enthalpy (Δ*H*_c_), and melting enthalpy (Δ*H*_m_) were determined from the second heating cycle to minimize the influence of prior thermal history [24]. The 14‐day time point was selected based on mechanical results showing no significant tensile changes beyond this period for most formulations. As noted earlier, thermal measurements were obtained from a single specimen per group; the following interpretation therefore addresses observed trends rather than statistically confirmed differences.

At baseline, *T*_g_ values for all materials fell within a narrow range (57.1–58.61°C), suggesting that filler incorporation produced only modest restrictions in chain mobility. The slightly higher *T*_g_ values observed for NaF‐ and GIC‐containing composites are consistent with the formation of interfacial regions that locally constrain polymer segment motion [33]. Within 14 days, *T*_g_ values appeared largely unchanged across groups, suggesting that major bulk thermal transitions occurred primarily after the initial 14‐day period for most formulations.

In contrast, crystallization‐related parameters showed greater sensitivity to composition and degradation. Pure PLA exhibited low initial Δ*H*_c_ and Δ*H*_m_ values, reflecting its inherently slow crystallization kinetics [34]. After aging, both enthalpies increased markedly, which may reflect enhanced crystallization associated with physical aging and hydrolysis‐induced chain scission—a process expected to increase chain mobility and facilitate molecular rearrangement into ordered domains [35–39].

For PLA/NaF composites, low NaF addition (5 wt%) increased Δ*H*_c_ and Δ*H*_m_ at baseline, consistent with heterogeneous nucleation effects reported for sodium salts in PLA matrices [34]. At higher NaF loadings, crystallization enthalpies were initially lower, suggesting that excessive filler content disrupted uniform chain alignment, resulting in less perfect or incompletely developed crystalline regions [40, 41], and showed nonuniform changes after aging. This pattern may reflect the combined effects of filler‐induced nucleation [34] and degradation‐driven heterogeneity arising from NaF dissolution during immersion [29, 30]. Localized degradation and structural irregularities could plausibly disrupt uniform chain organization [42], resulting in less predictable crystallization behavior at elevated NaF contents.

PLA/GIC composites showed a broadly comparable baseline response to PLA/NaF blends, with a low GIC content (5 wt%) substantially elevating both Δ*H*_c_ and Δ*H*_m_ through effective promotion of ordered crystallization [34]. Increasing the GIC content was associated with lower crystallization enthalpies, attributable to the same mechanisms previously discussed: excessive filler loading interfered with crystallite development [40, 41]. After aging, Δ*H*_m_ decreased for all GIC‐containing formulations, while Δ*H*_c_ declined progressively with increasing GIC concentration. Taken together, these observations suggest that although degradation can increase segmental mobility [43], high GIC loadings may impose geometric and physical constraints that limit chain reorganization during crystallization [40, 41]. These interpretations remain tentative given the descriptive nature of the thermal dataset and warrant confirmation through replicated measurements in future work.

### 4.3. Mechanical Property

The tensile behavior of pure PLA and PLA‐based composites reflected the combined influence of crystallization, degradation, and filler content. Pure PLA exhibited the highest initial tensile strength and showed a significant increase during the first 2 weeks, consistent with physical aging and increased crystallinity [44, 45]. The pronounced strength reduction observed at later time points indicates that progressive chain scission eventually outweighed the reinforcing effects of crystallization [45].

In NaF‐containing composites, tensile strength was strongly concentration‐dependent. The 5 wt% NaF formulation showed a transient increase in tensile strength, likely reflecting limited reinforcement and moderate crystallinity enhancement. At higher NaF contents, the tensile performance deteriorated earlier, particularly for the 20 wt% formulation. This behavior is consistent with SEM observations of increased void formation and DSC evidence of heterogeneous crystallization, both of which suggest the disruption of matrix continuity [46] and accelerated hydrolytic degradation promoted by NaF dissolution [29, 30].

PLA/GIC composites exhibited a comparatively stable tensile behavior. The 5 wt% GIC formulation showed a significant increase in tensile strength at 14 days, followed by stabilization, indicating an initial structural reorganization toward more load‐bearing domains [47, 48]. Higher GIC loadings did not significantly alter tensile strength over time, suggesting that excessive filler content neither enhanced reinforcement nor exacerbated degradation under the tested conditions. Overall, low‐level GIC incorporation provided a favorable balance between mechanical stability and degradation resistance.

Taken together, the SEM, DSC, and tensile strength results indicate that NaF primarily accelerates degradation through dissolution‐driven mechanisms, whereas GIC contributes to greater structural stability but limits crystallization and mechanical enhancement at higher concentrations.

## 5. Conclusion

PLA composites containing cured GIC exhibited more stable morphological, thermal, and mechanical behavior under oral‐simulating degradation conditions than those of NaF‐containing composites. Among the tested formulations, PLA containing 5 wt% GIC demonstrated the most balanced performance, maintaining relatively stable mechanical property while preserving crystallization behavior during aqueous aging, supporting its potential as a candidate fluoride‐releasing film for further preclinical evaluation in dental appliance applications, including denture bases, occlusal splints, and orthodontic retainers.

## Author Contributions

Conceptualization, methodology: Rungroj Kuson and Kullapop Suttiat. Data curation, investigation, visualization, writing – original draft: Rungroj Kuson. Formal analysis: Rungroj Kuson, Thanapat Sastraruji, and Kullapop Suttiat. Funding acquisition, project administration, resources, supervision: Kullapop Suttiat. Validation, writing – review and editing: Thanapat Sastraruji and Kullapop Suttiat. All authors made substantial contributions to the work reported, were involved in drafting, writing, or critical revision of the manuscript.

## Funding

This study was financially supported by the Faculty of Dentistry, Chiang Mai University, Chiang Mai, Thailand.

## Disclosure

All authors agreed to be accountable for all aspects of the work. All authors have read and approved the final version of the manuscript. The corresponding author and manuscript guarantor had full access to all of the data in this study and takes complete responsibility for the integrity of the data and the accuracy of the data analysis.

## Ethics Statement

This study was an in vitro laboratory investigation performed exclusively on synthetic and commercially available materials, namely, poly(lactic acid), sodium fluoride, glass ionomer cement, and PBS. It did not involve any human participants, human‐derived tissues or data, or animals. Ethical approval from an institutional review board or animal ethics committee was therefore not required.

## Conflicts of Interest

The authors declare no conflicts of interest.

## Data Availability

The data that support the findings of this study are available from the corresponding author upon reasonable request.
